# Risk of food insecurity and its association with social determinants of health among hospitalized patients in Lebanon

**DOI:** 10.1186/s40795-025-01196-x

**Published:** 2025-11-14

**Authors:** Lamis Jomaa, Joelle Abi Kharma, Nahla Hwalla, Emmanuel Kabengele Mpinga, Ngianga-Bakwin Kandala, Krystel Ouaijan

**Affiliations:** 1 Department of Nutrition, Gillings School of Global Public Health, University of North Carolina, Chapel Hill, USA; 2 Department of Nutrition and Food Sciences, Faculty of Agriculture and Food Sciences, American University of Beirut, Beirut, Lebanon; 3 Department of Nutrition and Food Sciences, Faculty of Arts and Sciences, Lebanese American University, Beirut, Lebanon; 4 Institute of Global Health, University of Geneva, Geneva, Switzerland; 5 Department of Epidemiology and Biostatistics, Schulich School of Medicine and Dentistry, Western University, London, ON Canada; 6 Division of Epidemiology and Biostatistics, School of Public Health, University of the Witwatersrand, Johannesburg, South Africa

**Keywords:** Food insecurity, Food security screening, Social determinants of health, Conflict-affected settings, Healthcare

## Abstract

**Background:**

Food insecurity is a growing concern globally, particularly in conflict-affected settings. In these contexts, hospitalized patients face heightened risks of poor health outcomes. The present study aims to assess the risk of food insecurity among hospitalized patients in Lebanon and investigate its association with social determinants of health (SDH) amidst multiple crises.

**Methods:**

A cross-sectional observational study was conducted from May to October 2021 on a random sample of adult hospitalized patients in five large hospitals across different districts inLebanon. A structured survey was used to collect socio-demographic characteristics, sources of health coverage, and medical history among study participants. In addition, survey included analysis of four indicators considered as integral part of SDH criteria: (1) area of residence and household size, (2) level of education, (3) employment status and type of employment, (4) healthcare access and coverage. Risk of food insecurity among praticipants was screened by a validated two-question tool, adapted from the US Department of Agriculture Household Food Security Survey. Associations between the SDH and risk of food insecurity were explored using logistic regression analysis using STATA V13.1.

**Results:**

Among the 343 participants, the majority (79.5%) were identified as being at risk of food insecurity with 62.4% classified as experiencing mild food insecurity, 15% as moderate, and 2.1% living with severe food insecurity. Higher odds of food insecurity were observed among residents of of predominantly rural areas mainly in the North of Lebanon (OR = 6.59, CI [1.79; 24.32], *p* = 0.005) and Bekaa (OR = 2.55, CI [0.92; 7.05], *p* = 0.071) districts. Additionally, participants with higher levels of education, particularly those with high school degree or higher, had lower odds of food insecurity (*p* < 0.05). Employment status, household size, and healthcare coverage were not found to be significant predictors of food insecurity among hospitalized patients in the multiple logistic regression analysis in the study sample.

**Conclusion:**

The study highlights the critical role of SDH, including educational level and geographical residence on experience of food insecurity among hospitalized patients. Screening for risk of food insecurity and associated determinants in health care settings are critical to design adequate programs and interventions to mitigate the risk of food and nutrition insecurity among vulnerable groups, particularly in conflict-affected settings.

## Introduction

Food insecurity, defined as the lack of reliable access to sufficient quantities of affordable, nutritious food, remains one of the pressing global concerns [1]. According to the State of Food Security and Nutrition in the World 2023 report, an alarming and significant increase in the prevalence of food insecurity has been observed over the past decade [2]. As of 2022, almost 29.6% of the global population – 2.4 billion people – were moderately or severely food insecure with higher rates of food insecurity reported among populations residing in low-to- middle income countries (LMICs) ranging between 28.7% and 58.3% compared to high-income countries (16%) using the Food Insecurity Experience Scale FIES [2].

Protracted conflicts, economic instability, climate change, and the prolonged ramifications of the COVID-19 pandemic have all contributed to the upward trend in food insecurity and hunger, particularly in low- and middle-income countries (LMICs) [3–7]. These challenges disproportionately impact vulnerable populations, including those living in poverty or conflict-affected areas, further amplifying the food insecurity crisis [8, 9]. The Middle East and North Africa (MENA) region remains one of the most food insecure regions in the world contributing to 20% of the world’s acutely food insecure population, a disproportionately high figure given its mere 6% share of the global population [10]. The high rates of food insecurity reported in the MENA region has been attributed to geopolitical factors, environmental stressors, high poverty, economic and social inequities, and protracted conflicts further threatening most vulnerable groups.

Food insecurity is a complex issue influenced by factors at the community, household, and individual levels, including low education levels, unemployment, and lower income levels. It is also associated with limited access to healthcare—both in terms of facility availability and coverage—and living in impoverished settings with inadequate infrastructure [11, 12]. These factors are also known as the Social Determinants of Health (SDH) and can impact health status of individuals and population groups, through influencing individual’s or household’s living and working conditions and their ability to access and consume sufficient, safe and nutritious food [13–16]. Despite the recognized importance of SDH, studies specifically linking these determinants to food security remain limited [14]. Most existing research has focused on individual factors such as economic status or gender, without examining these determinants collectively, as part of the broader SDH framework [17–19].

Food insecurity has also been studied amongst hospitalized patients, primarily by assessing caregivers of children during their hospital stay, with rates ranging between 5% and 25% among those screened [20, 21]. However, studies have been mostly limited to the pediatric population and have not linked risk of food security with SDH [20, 21]. Furthermore, there is a significant gap in evaluating whether hospitals are indeed an optimal setting for detecting food insecurity and their potential role in early screening and provision of support to individuals experiencing food insecurity, who might otherwise be difficult to reach in community settings [22, 23]. This gap in the literature underscores the need for comprehensive studies that explore how these interconnected social factors influence food security, while also assessing the effectiveness of hospital-based screening in addressing food insecurity [23, 24]. Targeting food insecurity in healthcare settings becomes particularly crucial in vulnerable settings where hospitalized patients face an increased risk of food insecurity and malnutrition due to the financial strain and resource limitations associated with their hospitalization [25, 26]. In contexts where health systems are overwhelmed, such as conflict-affected regions or resource-limited areas, the combined effects of poverty, displacement and inadequate healthcare exacerbate the risk of food insecurity for patients already vulnerable due to their health conditions [27, 28].

Lebanon is a small middle eastern country with acheckered history of wars, political and economic instability along with decades of poor governance. These conditions have led to an unprecedented financial crisis that has hit the country by end of 2019 and was excerberated later on by the COVID pandemic and its prolonged effects [29]. With a population of approximately 6.8 million, Lebanon hosts one of the highest refugee concentrations per capita in the world, sheltering over 1.5 million Syrian refugees and about 475,000 Palestinian refugees, as well as other forcibly displaced individuals from conflict-affected countries in the MENA region [30]. This influx of refugees has put significant pressure on Lebanon’s healthcare system, which has been severely strained over the years due to high demand from refugees and host communities. Additionally, Lebanon’s economy has been facing a catastrophic decline, with the Gross Domestic Product (GDP) contracting by over 58% between 2019 and 2021. This was considered one of the sharpest declines globally, accompanied by a severe devaluation of the Lebanese pound, which lost more than 90% of its value [29]. As a consequence of the economic collapse, approximately 40% of the population lost healthcare coverage leading to a sharp rise in out-of-pocket healthcare payments, with many individuals forced to prioritize medical expenses over food and other basic necessities [31, 32].

The present study aims to assess the risk of food insecurity among hospitalized patients in Lebanon and explore the relationship between SDH and food insecurity. The underlying hypothesis is that these social determinants, such as education, employment status, and access to healthcare, significantly influence food security, particularly in the context of Lebanon’s ongoing economic and political conflicts. By identifying the key predictors of food insecurity, this research seeks to inform policies and interventions aimed at addressing SDH, ultimately improving food security in conflict-affected settings and mitigating their adverse health impacts.

## Materials and methods

### Study population

Data for the present study was based on a cross-sectional, observational, multicenter study that explored the national prevalence of malnutrition among hospitalized patients in Lebanon [33]. The sample size for the original study was determined to estimate the prevalence of malnutrition using the STEPS sample size calculator of the WHO based on annual hospital admissions and it was estimated to be 330 hospitalized patients [34]. This number was chosen to achieve a 95% confidence interval with a margin of error of 0.05 and considering a significance level of 5% with 80% power.

**Fig. 1:**
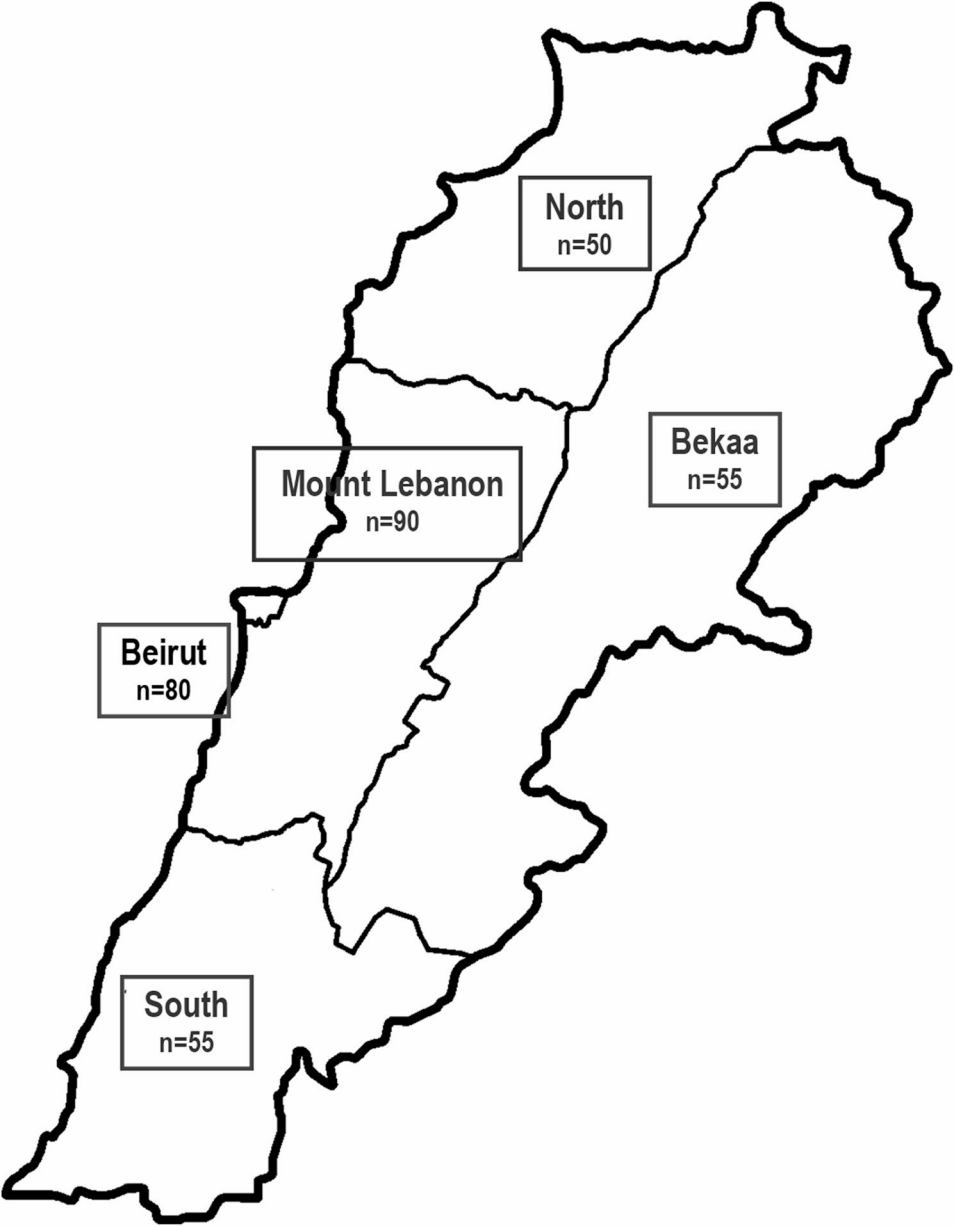
Distribution of sample according to district representation

Lebanon is divided into five districts, with the highest levels of urbanization found in Beirut and Mount Lebanon, while the Bekaa, North, and Tripoli regions are characterized by more rural structures and significant agricultural lands. A total of five hospitals, one hospital from each of the five districts of Lebanon, were selected by convenience sampling. The number of patients in the five different hospitals was weighed against the number of admissions per district from the Lebanese National Health Survey [35]. The distribution of sample according to districts, to have a national representation, is presented in Fig. 1.

All adult patients, both males and females aged 18 years and above, admitted to various wards of the hospital during the data collection period were recruited within 48 h of admission. The exclusion criteria were patients admitted to gynecology department for delivery and pregnancy-related complications, intensive care units, psychiatry wards, and those with a short stay of less than 48 h.

### Data collection and social determinants of health

Data collection took place from May to October 2021. A survey consisting of 21 questions covering socio-demographic characteristics, sources of health coverage, and medical history was administered to all participants who consented to take part in the study.The survey was administered by hospital dietitians, who had been trained to conduct the survey and relaying questions accurately in Arabic (official language in Lebanon and mother tongue) to all study participants.

The survey included questions pertinent to patient’s basic characteristics, including age, gender, marital status, admission diagnosis and history of previous admissions were recorded. In addition, the survey included four indicators considered as integral part of Social Determinants of Health [36, 37]:

1. Area of Residence and Household Size: Evaluated under neighborhood and urbanization.
2. Level of Education: Assessed to understand educational attainment.
3. Employment Status: Classified under economic stability.
4. Source of Health Cost Coverage: Used as a metric for measuring healthcare access.

Area of residence included the five districts of Lebnaon. The household size was dichotomized into two groups (less than four members) and greater than four members, which is considered the average household size for Lebanon [38]. Employment status was categorized to include part-time, full-time, self-employed, and retired individuals, as all these groups have a source of income, whether through wages, self-employment earnings, or pensions. Level of education was presented as starting from no schooling up to university education and included three groups: intermediate or less, high school or technical diploma, and university degree. Sources of health insurance coverage that apply to the country context included the National Social Security Fund (NSSF), private insurance and financial aids from governmental and non-governmental organizations. Income level could not be included in the survey due to the recent depreciation of the national currency. The rapid depreciation made traditional income categories unreliable and unable to reflect economic status accurately.

### Food insecurity

Risk of food insecurity at household level was screened using a screening tool adapted from the 2000 United States Department of Agriculture Report on Food Security Measurement Project [21, 39–42]. Using a two-item screening tool with different combinations of questions from USDA tool was previously validated in healthcare settings [21, 41, 42]. If an affirmative response was given to either of the two questions, the individual was considered at risk for food insecurity. In this study, we specifically selected questions HH1 and HH4 from the USDA survey, incorporating them into the broader survey used in the national study [21, 27, 39]. The first question, which reflects “food quantity”, was “Which of these statements best describes the food eaten in the household in the last 12 months?” The response categories were (1) enough of the kinds of food we want to eat, (2) enough but not always the kinds of food we want, (3) sometimes not enough to eat and (4) often not enough to eat. The second question, which we refer to as “food quality”, was “Which of these statements best describes the quality of food eaten in the household in the last 12 months?” The response categories were (1) very good, (2) good, (3) average and (4) poor. If options 2, 3, or 4 were selected for either or both of the two questions, it was considered an affirmative response, and the individual was categorized as at risk for food insecurity. Furthermore, the food insecurity at risk category was further classified as (2) mild, (3) moderate, or (4) severe, depending on the responses to one or both questions.

A conceptual framework was developed to illustrate the overall approach by mapping the hypothesized relationships between the Social Determinants of Health and the risk of food insecurity among hospitalized patients.

### Statistical analysis

Statistical analysis was performed using STATA V13.1. Descriptive analysis was used to summarize the study variables and to check for out of range values. Continuous variables were presented using means and standard deviations, while frequencies and percentages were used to represent categorical variables. The normality of the data was assessed using the Shapiro-Wilk test. For the non-parametric variable age, the median was used to dichotomize the variable using the cutoff point of 60 years old to align with the standard classification for older adults as defined by the World Health Organization (WHO). A series of simple logistic regressions were conducted at the bivariate level to identify potential correlates of food insecurity. Participants who provided at least one mild, moderate, or severe answer to either of the questions were labeled as “at risk of food insecurity,” while those with two normal answers were labeled as “not at risk.” Variables for inclusion in the multivariable model were selected based on contextual relevance and a p-value < 0.1 at the bivariate level. A multiple logistic regression was conducted thereafter to etimate odds ratios (ORs) and 95% confidence intervals (CIs) with food insecurity serving as the primary dependent variable and the independent variables included in the model were sociodemographic characteristics and other SDH variables. The overall models’ goodness-of-fit was assessed using Hosmer-Lemeshow’s goodness-of-fit statistics. A p-value that exceeds 0.05 indicates a good model fit [43].

### Ethical approval

The study was completed in compliance with the guidelines of the Helsinki Declaration. The study protocol was reviewed and approved by the Institutional Review Board of the American University of Beirut (SBS-2020-0079). Approval for data collection from the Ministry of Health and from the administration of each hospital were secured prior to initiation of data collection. All participants reviewed and signed an informed consent form before participation.

## Results

### Baseline characteristics

A total of 343 participants were enrolled in the study. All participants responded to the survey on food security and social determinants of health. Baseline characteristics are presented in Table 1. The majority of participants were male (54.8%), married (70.5%), and of Lebanese nationality (92.4%). Additionally, 68.8% of participants had at least one chronic condition in their medical history, with diabetes, hypertension, and dyslipidemia being the most prevalent. Only 14% of the participants reported a prior hospital admission within the past three months.

**Table 1:** Demographic characteristics, admission diagnosis and medical history of participants (*n* = 343)

	*n* (%)
Age	
< 60 years old	155 (45.2%)
≥ 60 years old	188 (54.8%)
Gender	
Male	188 (54.8%)
Female	155 (45.2%)
Marital Status	
Married	242 (70.5%)
Not Married	101 (29.5%)
Country of Origin	
Lebanon	317 (92.4%)
Syria	12 (3.5%)
Palestine	11 (3.2%)
Others	3 (0.9%)
Medical charateristics	
Admission diagnosis	
Oncology	25 (7.3%)
Heart disease	53 (15.4%)
Infectious disease	95 (27.7%)
Gastrointestinal disease	40 (11.7%)
Surgery	113 (32.9%)
Other	17 (5.0%)
Past Medical History	
None	87 (25.4%)
Chronic Diseases^†^	236 (68.8%)
Others^‡^	20 (5.8%)
Hospital Admission in 3 months	
Yes	48 (14.0%)
No	295 (86.0%)

^†^Diabetes, hypertension, dyslipidemia, cancer; ^‡^ Neurological disorders, gastrointestinal diseases

### Social determinants of health

Based on the four indicators of social determinants, the results are presented in Table 2. When studying area of residence and household size, most individuals resided in Mount Lebanon (29.2%), Beirut (23.6%) and the South (16.9%). The average household size was 3.58 (SD = 1.64), with 46.4% of households having four or more members. As for level of education, 28.0% of study participants hold university degrees, 26.2% had high school diplomas and 45.8% had intermediate level or less. As a measure of economic stability, the employment status was as follows: 45.8% of participants were unemployed, 54.2% were either employed full-time, part time or retired. Healthcare access, measured by types of health coverage, was distributed among NSSF or support from army and other government institutions (49.0%) and private insurance (36.1%). A smaller percentage of 14.9% of the population had no health coverage at all and rely on NGO funds when needed.

**Table 2:** Indicators for social determinants of health among participants (*n* = 343)

	*n* (%)
Neigbourhood	
District	
Beirut	81 (23.6%)
North	50 (14.6%)
South	58 (16.9%)
Mount Lebanon	100 (29.2%)
Bekaa	54 (15.7%)
Household Size	
< 4 members ≥ 4 members	184 (53.6%) 159 (46.4%)
Education	
Level of Education	
Intermediate or less	157 (45.8%)
No schooling	32 (9.3%)
Primary school	59 (17.2%)
Intermediate school	66 (19.3%)
High school/Technical diploma	90 (26.2%)
University degree	96 (28.0%)
Employment	
Work Status	
Unemployed	157 (45.8%)
Employed/Retired	186 (54.2%)
Employed full time	91 (26.5%)
Employed part time	11 (3.2%)
Self-employed	29 (8.4%)
Retired	55 (16.1%)
Healthcare Access	
Health Cost Coverage	
None/NGOs†	51 (14.9%)
NSSF‡/Army	168 (49.0%)
Private insurance	124 (36.1%)

†NGO: non-governmental organizations; ‡NSSF: National Social Security Fund

### Risk of food insecurity

79.3% (*n* = 272) of participants were identified as being at risk of food insecurity with 62.4% experiencing mild food insecurity, while 14.9% and 2.0% were living with moderate to severe food insecurity, respectively. Regarding the quantity of food consumed, more than half of the study participants (58.3%) reported that their household food was “enough but not always the kinds of food we want.” Only 6 participants (1.7%) indicated that they did not have enough food to eat. When assessing the quality of food in the household, responses were mainly split between “good” (32.4%) and “average” (38.2%). Results are represented in Table 3.

**Table 3:** Risk of food insecurity and distribution of risk categories among study participants (*n* = 343)

	*n* (%)
Risk of Food Insecurity	
Normal	71 (20.7%)
At risk	272 (79.3%)
Food Insecurity Risk Categorization	
Normal	71 (20.7%)
Mild	214 (62.4%)
Moderate	51 (14.9%)
Severe	7 (2.0%)
Q1: Food Quantity	
Enough of the kinds of food we want to eat	87 (25.4%)
Enough but not always the kinds of food	200 (58.3%)
Sometimes not enough to eat	50 (14.6%)
Often not enough to eat	6 (1.7%)
Q2:Food Quality	
Very good	80 (23.3%)
Good	111 (32.4%)
Average	131 (38.2%)
Poor	21 (6.1%)

### Bivariate association analysis: risk of food insecurity with social determinants of Health, hospital Readmission, and medical history

Factors associated with the risk of food insecurity were level of education, geographical location, and healthcare coverage as shown in Table 4. Specifically, residents in the North (*p* = 0.001), Mount Lebanon (*p* = 0.043) and Bekaa (*p* = 0.007) districts had higher odds of being identified as at risk of food insecurity compared to those in Beirut. Participants holding high school or technical degrees (*p* = 0.020) and with university degrees (*p* = 0.001) displayed lower odds of being identified at risk compared to those with intermediate degrees or lower. Participants covered by NSSF and the army exhibited increased risk of food insecurity compared to those covered by private insurance (*p* = 0.020).

Two additional factors, previous hospital admission and medical history were included in the bivariate analysis. Among those identified as at risk of food insecurity, only 15.1% had been hospitalized in the past three months, compared to almost 10.0% of those not at risk of food insecurity. There was no significant association between food insecurity and hospital admissions (*p* = 0.263), nor between food insecurity and past medical history (*p* = 0.989) during this period.

### Multiple logistic regression analysis: associations between risk of food insecurity with SDH correlates

Level of education and district were found to be significant correlates of the risk of food insecurity as shown in Table 4. Specifically, participants with university degrees had lower odds of food insecurity compared to those with intermediate degrees or lower (adjusted OR = 0.32, 95% CI 0.15–0.70, *p* = 0.004). Participants residing in the North and Bekaa had higher, and borderline higher odds of food insecurity compared to those residing in the capital, Beirut (Adjusted OR = 6.59, 95% CI 1.79–24.32; *p* = 0.005 and adjusted OR = 2.55, 95% CI 0.92–7.05, *p* = 0.071 respectively). The Hosmer and Lemeshow’s goodness-of-fit test indicate that our model fit the data well with a p-value of 0.2358.

**Table 4:** Bivariate and multivariable logistic regression analyses exploring associations between food Insecurity, social determinants of Health, hospital Readmission, and medical history

Variable	Bivariate Analysis		Multivariable Logistic Analysis*	
	Odds Ratio (95% CI)	*P*-value	Odds Ratio (95% CI)	*P*-value
*Age*				
< 60 years (*Reference*)				
≥ 60 years old	0.92 (0.55; 1.56)	0.772	0.60 (0.32; 1.12)	0.113
*Gender*				
Male (*Reference*)				
Female	1.34 (0.78; 2.29)	0.275	1.30 (0.66; 2.58)	0.431
*Employment status*				
Not Employed (*Reference*)				
Employed/Retired	0.62 (0.36; 1.06)	0.084	0.82 (0.41; 1.67)	0.597
*Level of Education*				
≤ Intermediate school (*Reference*)				
High school/Technical diploma	0.45 (0.23; 0.88)	0.020*	0.51 (0.24; 1.07)	0.078
University degree	0.34 (0.18; 0.63)	0.001*	0.32 (0.15; 0.70)	0.004*
*Marital Status*				
Single/Divorced/Widowed(*Reference*)				
Married	0.70 (0.38; 1.28)	0.255	-	-
*District*				
Beirut (*Reference*)				
North	8.27 (2.36; 28.99)	0.001*	6.59 (1.79; 24.32)	0.005*
South	2.02 (0.92; 4.43)	0.077	1.03 (0.39; 2.74)	0.943
Mount Lebanon	1.98 (1.02; 3.86)	0.043*	1.66 (0.83; 3.34)	0.154
Bekaa	3.55 (1.42; 8.87)	0.007*	2.55 (0.92; 7.05)	0.071
*Household size*				
< 4 individuals (*Reference)*				
≥ 4 individuals	1.32 (0.78; 2.25)	0.297	-	-
*Health Coverage*				
Private insurance (*Reference)*				
None/NGOS	1.54 (0.69; 3.43)	0.281	1.01 (0.39; 2.57)	0.983
NSSF/Army	1.97 (1.12; 3.49)	0.020*	1.37 (0.69; 2.68)	0.359
*Hospital admission in past 3 months*				
Yes (*Reference)*				
No	0.61 (0.26; 1.43)	0.263	-	-
*Past Medical History*				
None (*Reference)*				
Chronic diseases	0.99 (0.54; 1.82)	0.989	-	-
Others ‡	1.04 (0.31; 3.50)	0.945	-	-

*Multivariable logistic regression models were adjusted for age, gender, employment status, level of education, district, and type of health coverage

† Diabetes, hypertension, dyslipidemia, cancer

‡ Neurological disorders, gastrointestinal diseases

## Discussion

The present study examined the risk of food insecurity among hospitalized patients in Lebanon and their association with key SDH variables.To our knowledge, this is the first study that explores the risk of food insecurity at a household level among hospitalized patients in a fragile context in the MENA region.

Overall, a high proportion of our study participants were identified as at risk of food insecurity. These results corroborate the worrisome increase in the prevalence of food insecurity reported in studies published pre- and post the COVID-19 pandemic and the economic crisis that Lebanon has been witnessing over the last five years [44–46]. In 2019, Kharroubi and colleagues [44] estimated the impact of the pandemic and economic crises on food insecurity prevalence in Lebanon using data from the Gallup World Poll (GWP) and while considering different income scenarios and projections. Pre crises, trend analyses showed that food insecurity was around 27%, while post crises prevalence of food insecurity were estimated to reach on average 36% to 39% of the country’s population, considering 50–70% income reduction scenarios [44]. A more recent study conducted in 2021 on 1,133 participants from different districts in Lebanon to assess levels of food insecurity among households using mobile application found that 75.4% of households were severely food-insecure with half of population studied reporting low food consumption scores and coping strategies like skipping meals or compromising food quality [45]. Similarly, a 2022 online survey targeting parents of school-aged children reported food insecurity in 75% of households amidst the ongoing crisis [47]. Although previous studies have used different tools that may be more comprehensive or have shorter recall periods, the findings from the present study remain worrisome given the high risk of food insecurity reported amongst acutely admitted hospital patients in the present study.

The present study also examined the associations between risk of of food insecurity and SDH variables. When exploring the household and neighborhood factors within SDH, it was observed that household size did not significantly impact the risk of food security, especially considering that the household size in our study population was close to the national average. However, the area of residence was found to be a significant correlate of food insecurity in the adjusted model. More specifically, participants from the more rural North and Bekaa regions faced significantly higher odds of being at risk of food insecurity—6.28 and 2.62 times higher, respectively, than those in the capital Beirut—though the wide confidence interval for the North, likely due to a smaller sample size, warrants cautious interpretation of this finding. This association highlights a finding consistent with studies in other regions of the world, where urbanization is frequently linked to improved food security, largely due to better access to markets and employment opportunities mainly in low and middle income countries [48].​ Conversely, participants from regions of North and Bekaa, which are traditionally agricultural lands, were significantly associated with higher risk of food insecurity. Although residents in rural areas may have potential food availability and access to land, their purchasing power has been reported to be lower than those in urban and suburban areas with better employment rates and higher incomes, thus better economic access to food [49, 50]. Other factors that can contribute to the higher risk of food insecurity among rural residents, and as per the scientific literature, are potential challenges within the food supply chain, such as inadequate infrastructure and poor distribution networks which can limit the availability and affordability of nutritious food even in areas where food production is prevalent​ [49, 51, 52].

Another significant correlation found in the present study was between educational attainment, a key SDH, and food security. More specifically, higher levels of education were significantly associated with a lower risk of food insecurity, while employment status was not found to be a significant correlate with the risk of food insecurity among student participants. Educational attainment has been well established in the literature as a predictor of food insecurity at both the individual and household levels [53–55]. This finding also aligns with several studies conducted in Lebanon, and similar fragile settings [56–58], showing that households with higher education levels were significantly less likely to be food insecure compared to those with lower parental education levels. A former study in Kenya similarly found that education significantly influenced food security even after adjusting for household wealth, aligning with our findings that low education level, rather than employment, remained significant predictor of food insecurity in the multiple regression analysis [59]. The independent effects of education on food security may be also influenced by unobserved factors, such as resource allocation practices and knowledge to effectively meet the nutritional needs of the household, which play a crucial role in determining and maintaining food security [60, 61].

In the present study, the healthcare cost coverage emerged as a significant factor showing an association with food insecurity in the bivariate analysis. Although this association was no longer significant in the multiple regression model, it remains an important social determinant to consider when assessing food security. Access to health care and the extent of coverage for chronic diseases is an important determinant of health that enables beneficiaries to access necessary healthcare services while reducing out-of-pocket health expenditures and improves health outcomes [31, 62, 63]. This is particularly important in the context of refugees, who typically lack formal healthcare coverage and often rely on funding from non-governmental organizations (NGOs) for access to medical services, further exacerbating their vulnerability. Access to health care allows for financial stability and a greater allocation of resources towards other basic needs including securing adequate and nutritious food, ultimately improving food and nutrition security [64, 65].

Our study also highlights the importance of hospital settings and healthcare services as a valuable opportunity for early screening of food insecurity risks. Hospitals and healthcare services present an ideal setting for detecting individuals at risk of food insecurity, as patients already interact with healthcare providers and can be systematically screened [22]. The development of two-item screening tools, which have been validated in various healthcare settings in LMIC and high income settings, has also proven effective in identifying food insecurity [21, 41, 42, 66]. Such tools that are designed for screening purposes are particularly well-suited for healthcare settings due to their simplicity and ease of use, which require minimal time and training for implementation. Food insecurity screening tools can be seamlessly introduced into routine healthcare assessments, thus allowing healthcare providers to detect the risk of food insecurity early on and connect patients in need with the appropriate food assistance and social welfare programs required upon discharge [40, 66]. Such programs can also be customized to account for both the patients’ health conditions and their food insecurity status, ensuring interventions are designed to meet their specific nutritional needs and support long-term health outcomes [67]. Indeed, patients who are screened for food insecurity may be at a heightened risk of malnutrition and other health complications, due to the limited resources and high out of pocket medical expenses in Lebanon which places them at higher risk for hospital readmission and additional financial burdens [63]. By integrating food assistance with medical care, these interventions can help improve overall patient wellbeing while also reducing the burden on healthcare systems, which is essential in settings with strained health care systems [68].

Recently, initiatives such as the food is medicine (FiM) approach has been gaining momentum in high income countries such as the United States whereby the role of food is emphasized as a key component of healthcare interventions for promoting food and nutrition security among vulnerable populations [69]. These FiM programs are designed to address both acute and chronic health conditions through medically-tailored food interventions and other produce prescription modalities [67, 69, 70]. The emerging literature that explores health care–based food insecurity interventions is rather promising, however there is still limited evidence on the effectiveness of these interventions on food insecurity and other nutrition and health-related outcomes [70]. Nevertheless, this is an area of research that is worth further exploring, particularly in conflict-affected areas and settings, undergoing the multiple burden of food insecurity, malnutrition and poor health outcomes.

**Fig. 2:**
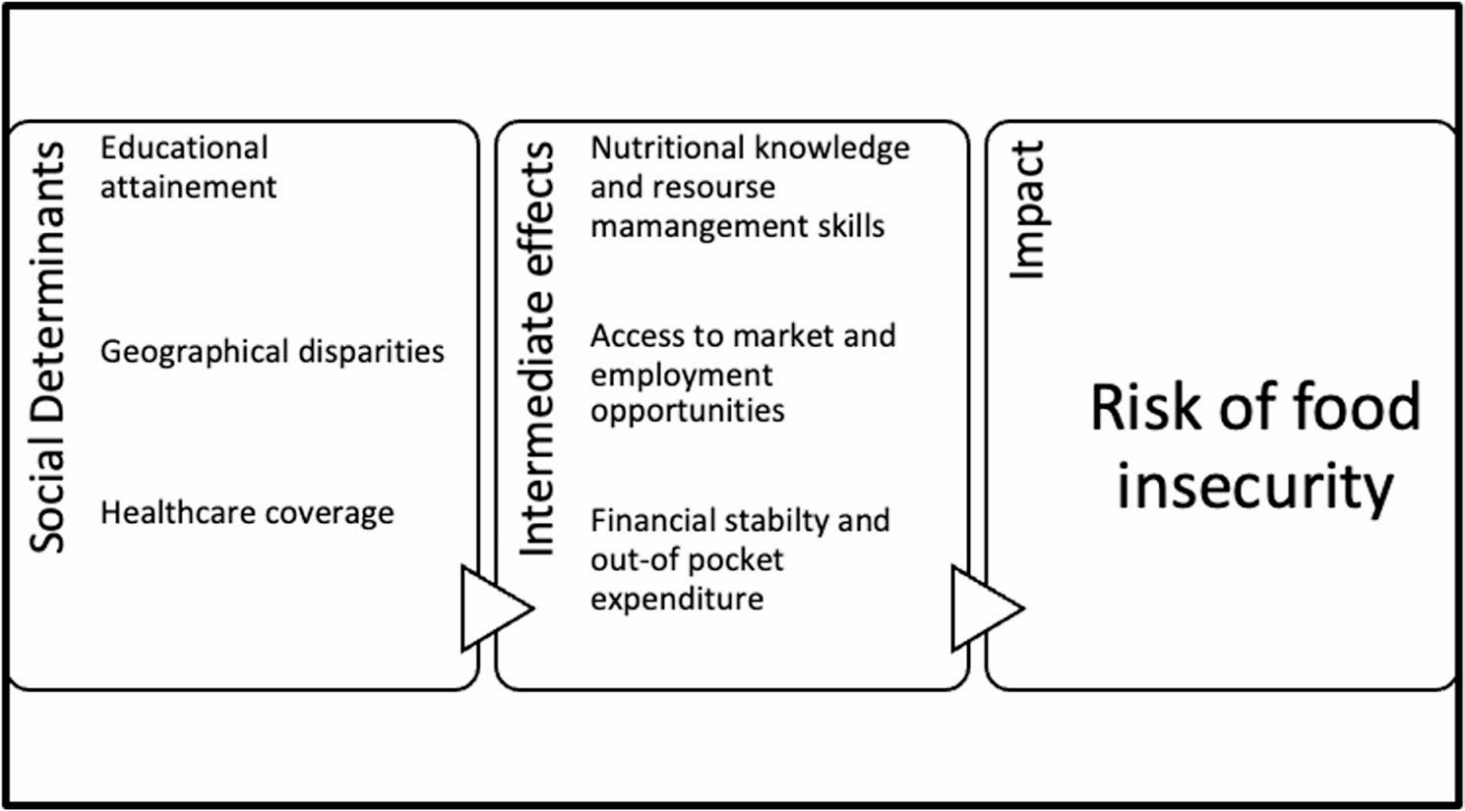
Proposed Conceptual Framework on Social Determinants of Health an and Risk of Food Insecurity

Our study findings highlight the critical role of social determinants of health (SDH) on the risk of food insecurity, in influencing food security. The proposed conceptual framework has been adapted from the WHO framework on SDH [16] illustrating the interplay between social determinants of health (education, geographic location, and healthcare access) with their intermediate effects on the risk food insecurity which can then contribute to a host of adverse health outcomes (see Fig. 2). In fact, inadequate education limits resource management skills, geographic disparities hinder access to food markets and employment opportunities, and insufficient healthcare coverage affects financial stability—all of which exacerbate food insecurity, particularly in fragile and conflict-affected settings where needs are more pronounced [18, 19].

### Limitations and strengths

One of the key strengths of this study is its focus on hospitalized patients in a fragile country considered as an emergency-affected setting, providing valuable insights into the risk of food insecurity and its association with SDH. The study’s multicenter approach ensured a diverse sample from different geographical areas and hospital settings across the country, thus enhancing the generalizability of the findings. Additionally, it is one of the few studies utilizing a validated two-item tool for food security screening in hospitalized settings, contributing to the accurate identification of food insecurity among this vulnerable population. Nevertheless, the study has several limitations that are also worth considering. One limitation is the cross-sectional nature of the study, which limits our ability to explore causal relationships between SDH and food insecurity. Respondent bias cannot be elimited in the present study, as social and economic indicators were collected from patients and their caregivers with some respondents expressing reservations when answering certain questions, potentially affecting the accuracy of the data. Additionally, due to the fluctuation of the national currency and its remarkable currency devaluation during the time of the study, questions relevant to income and financial resources and assets were not explored, which limited the analysis of income as part of social determinants in relation to food security. Furthermore, as the study focused on hospitalized patients, it is important to acknowledge that while this population includes diverse groups and represents various segments of society, it should not be considered fully representative of population-level estimates, especially since only private hospitals were included due to restricted access to public hospitals during the data collection period.

## Conclusion

The study highlighted the critical role of SDH on risk of food insecurity among hospitalized patients in Lebanon. Findings from the present study highlight the critical role that hospitals and other health care settings can provide for early screening and detection of food insecurity. Hospitals can serve as a setting to provide at-risk individuals with access to resources and services including food assistance and social welfare programs that can help mitigate food and nutrition insecurity and reduce their risk of malnutrition and other adverse health outcomes.

## Data Availability

No datasets were generated or analysed during the current study.

## Abbreviations

SDH: Social Determinants of Health
FI: Food Insecurity
USDA: United States Department of Agriculture
NSSF: National Social Security Fund
NGO: Non-Governmental Organization
GDP: Gross Domestic Product
LMIC: Low- and Middle-Income Countries
